# Relationship between anxiety symptoms and cervical motor control in individuals without diagnosed psychiatric or neurological disorders

**DOI:** 10.3389/fpsyg.2026.1743293

**Published:** 2026-02-25

**Authors:** Andrea Calleja-Caballero, Juan Luis Sánchez-González, Marta Gómez-Mateos, Vanesa Santos-Rodríguez, Jesus Perez, Fátima Pérez-Robledo

**Affiliations:** 1 Department of Nursing and Physiotherapy, University of Salamanca, Salamanca, Spain; 2 Institute of Biomedical Research of Salamanca (IBSAL), Salamanca, Spain; 3 Department of Psychiatry, Faculty of Medicine, University of Salamanca, Salamanca, Spain; 4 Department of Psychiatry, University of Cambridge, Cambridge, United Kingdom; 5 Cambridgeshire and Peterborough NHS Foundation Trust, Cambridge, United Kingdom; 6 Norwich Medical School, University of East Anglia, Norwich, United Kingdom

**Keywords:** anxiety, cervical motor control, headache, neck pain, vertigo

## Abstract

**Objectives:**

This study aimed to explore the association between anxiety symptoms and cervical motor control in individuals without diagnosed psychiatric or neurological disorders.

**Methods:**

A cross-sectional study was conducted with 101 participants aged 18–60 without diagnosed psychiatric or neurological disorders. Anxiety levels were assessed using the Hamilton Anxiety Rating Scale (HAM-A), a clinician-oriented measure applied here to a non-clinical sample, and severity cut-offs were interpreted cautiously. Cervical motor control was measured using the Head Repositioning Accuracy-to-Target test. Additional clinical variables such as vertigo, cervical pain, and headache were also recorded. Statistical analyses included Spearman correlations, multiple linear and logistic regressions.

**Results:**

Higher anxiety levels were significantly associated with increased angular error in cervical motor control, particularly in flexion, extension, and right rotation movements. A progressive increase in pain perception and motor dysfunction was observed in participants with moderate and severe anxiety. Multivariate analyses showed that cervical motor control errors and vertigo were independently associated with anxiety severity and clinically significant anxiety.

**Conclusion:**

Our findings revealed an association between anxiety symptoms and cervical sensorimotor disturbances in individuals without diagnosed psychiatric or neurological disorders. Given the cross-sectional design, these findings should be interpreted as observational and exploratory.

## 1 Introduction

Anxiety disorders are usually characterized by anticipatory distress, uncertainty and difficulty in regulating fear-related responses (American Psychological Association [APA], 2018). They may also present with a wide range of somatic symptoms, such as cardiovascular and gastrointestinal disturbances, and musculoskeletal pain. In fact, musculoskeletal pain, specifically neck pain, is highly prevalent in such disorders (Mallorquí-Bagué et al., 2016; Zugman et al., 2021).

Neck pain involves discomfort localized in the cervical region, often with stiffness and a limited motion range. It represents one of the leading causes of disability and reduced quality of life worldwide. In terms of etiology, neck pain is multifactorial, involving biological, psychological and social determinants (Genebra et al., 2017). Non-modifiable risk factors include advanced age, female sex, and a prior history of cervical pain (Kazeminasab et al., 2022). Physical inactivity, psychosocial stressors and impairments in cervical motor control have been identified as significant contributors to both the onset and persistence of neck pain. Motor control impairments have been proposed as a key cause in the recurrence and chronicity of such pain. Prolonged deficits in proprioception - the body’s ability to sense movement and position - may induce neuroplastic changes that disrupt sensorimotor integration and maintain cervical pain (Brumagne et al., 2019). Patients with neck pain frequently have an altered cervical sensorimotor function. In this context, compensatory processes have been proposed, including increased activity of superficial muscles, delayed activation of deep stabilizing muscles, prolonged effort duration, and excessive co-activation as a protective mechanism, resulting in increased cervical stiffness (Gizzi et al., 2015; Tsang et al., 2014). Neck pain has been associated with heightened activation of mechanoreceptors that play a crucial role in postural control and head-eye coordination, resulting in a large amount of inaccurate sensory signals (Peng et al., 2021). These abnormal signals from the cervical region may disrupt the normal integration of sensory input from the visual and vestibular systems, leading to a sensory mismatch and, subsequently, cervicogenic vertigo (Li et al., 2022). These mechanisms have been primarily described in populations with chronic neck pain; however, it remains unclear whether similar, albeit subtler, cervical sensorimotor alterations may also be present in individuals without diagnosed pain or neurological disorders, particularly in relation to emotional factors such as anxiety.

Recent studies in clinical populations have shown that higher anxiety levels are associated with greater cervical pain severity. In fact, anxiety often overlaps with chronic pain conditions, ranking as the second most common comorbidity associated to neck pain (Hogg-Johnson et al., 2008). The bidirectional nature of this association suggests that anxiety may exacerbate neck pain through increased muscle tension and altered pain perception, whilst neck pain may also intensify anxiety symptoms (Genebra et al., 2017; Tanguay-Sabourin et al., 2023).

While this association has been well documented in clinical populations, less is known about how anxiety relates to cervical sensorimotor function in non-clinical individuals without diagnosed psychiatric or neurological disorders. It remains unclear whether anxiety symptoms are associated with measurable alterations in cervical motor control and related sensorimotor symptoms, such as dizziness, before the onset of clinically significant anxiety or chronic pain. Addressing this gap may help to clarify whether anxiety is linked to early or subclinical sensorimotor changes beyond pain-related mechanisms.

Based on this framework, the present study aimed to explore the association between anxiety symptoms and cervical motor control in individuals without diagnosed psychiatric or neurological disorders. We hypothesized that higher levels of anxiety symptoms would be associated with poorer cervical motor control performance. Additionally, we explored whether cervical sensorimotor alterations and related clinical symptoms, such as vertigo, were independently associated with anxiety severity.

## 2 Materials and methods

### 2.1 Study design

To achieve our aim, we conducted a descriptive, observational, cross-sectional study reviewed and approved by the Ethics Committee of University of Salamanca (Record number: 1376). All study procedures were conducted following the last revision of the Declaration of Helsinki (World Medical Association, 2001).

### 2.2 Study participants

Community-dwelling individuals without diagnosed psychiatric or neurological disorders were recruited using convenience sampling, through a recruitment strategy based on informational posters. The posters were distributed by the Faculty of Nursing and Physiotherapy at the University of Salamanca, Salamanca, Spain, and a CrossFit-style gym in the same city. They included a brief description of the study and a QR code that redirected them to a Google Calendar where they could select the available time slots to be assessed. In addition, all individuals had to meet the inclusion criteria, i.e., age between 18 and 60 years old and not being on anxiolytic or antidepressant medication; and the exclusion criteria, i.e., to have a known neurological or vestibular condition, and/or be unable to understand or sign the informed consent form.

### 2.3 Sample size

The study was conducted in a sample of 101 participants, drawn from an estimated target population of 23,000 adults (aged 18 and over) in the city of Salamanca who might eventually present characteristics compatible with a risk of cervical motor control deficit. The population size was estimated based on municipal demographic data (Intituto Nacional de Estadistica, 2024) and epidemiological literature reporting a prevalence of cervical dysfunction-related symptoms in approximately 20%–30% of the adult population (Fejer et al., 2006).

With these values, the margin of error associated with the sample size is approximately ±9.4%, which is considered adequate for observational and exploratory studies on variables related to neck pain, anxiety, and stress. This sample size was considered adequate for exploratory observational analyses and for identifying potential associations between anxiety symptoms and cervical motor control variables, in line with previous methodological recommendations for exploratory studies (Brysbaert, 2019).

### 2.4 Evaluations

Each participant was invited individually to a room set up for testing, under controlled environmental conditions (lighting, temperature, noise, etc.). An identification code was assigned to preserve participant anonymity and ensure confidentiality. Following this, a structured assessment was conducted, comprising three sections: (1) an information sheet describing the study, including a digital informed consent form; (2) a structured set of questions gathering relevant sociodemographic data (e.g., age, gender, level of physical activity); and (3) the Hamilton Anxiety Scale (HAM-A).

#### 2.4.1 Anxiety assessment

The Hamilton Anxiety Scale (HAM-A) (Hamilton, 1959) was used to measure anxiety symptoms. HAM-A has shown good psychometric properties, including adequate interrater reliability (Clark and Donovan, 1994; Maier et al., 1988), internal consistency (Clark and Donovan, 1994), and convergent validity (Beck et al., 1988). It is a questionnaire of 14 items to evaluate severity of anxiety symptoms, including both their psychological (cognitive and emotional) and somatic (physical) clinical dimensions:

- Psychological anxiety (7 items): (1) anxious mood, (2) tension, (3) fears, (4) insomnia, (5) alterations in intellectual functions (difficulties in concentration and memory), (6) depressive mood, and (14) behavior observed during the interview.
- Somatic anxiety (7 items): (7) somatic muscular symptoms, (8) sensory symptoms, (9) cardiovascular symptoms, (10) respiratory symptoms, (11) gastrointestinal symptoms, (12) genitourinary symptoms, and (13) autonomic nervous system symptoms.

Each item is scored from 0 to 4, where 0 indicates no symptoms and 4 indicates very intense symptoms. Regarding the interpretation of the total score, and even though there are no strict limits, the following approximate cut-off points are commonly used to estimate the degree of anxiety:

- <17 points: mild anxiety or within normal ranges.
- 18–24 points: moderate anxiety.
- 25–30 points: moderate to severe anxiety.
- >30 points: severe anxiety.

The Hamilton Anxiety Rating Scale (HAM-A) was administered by the investigator during the assessment session. Items 1–13 were completed with the active participation of the participant, while item 14 (behavior observed during the interview) was rated directly by the investigator based on clinical observation. Although the HAM-A was originally developed as a clinician-rated instrument for clinical populations, in this study it was used to assess anxiety symptom severity in a non-clinical sample. Therefore, cut-off scores were interpreted cautiously as indicative severity levels rather than diagnostic criteria.

#### 2.4.2 Cervical motor control assessment

Cervical motor control was assessed using the Head Repositioning Accuracy-to-Target (HRA-to-Target) test (Michiels et al., 2013).

Each participant was seated on a chair with back support, positioned approximately 1 m from a flat wall where a visual reference (not used during testing) was placed to standardize the environment. Participants adopted an upright and relaxed posture, with feet on the ground, pelvis in a neutral position, and arms resting on their legs. Their heads started from a neutral position in the sagittal plane. To eliminate visual input and enhance proprioception, participants were instructed to keep their eyes closed throughout the test. The evaluator then passively guided the participant’s head into 30° of cervical flexion and held this position for a few seconds. This maneuver was repeated three consecutive times to allow a somatic integration of the target position (Loudon et al., 1997).

**FIGURE 1:**
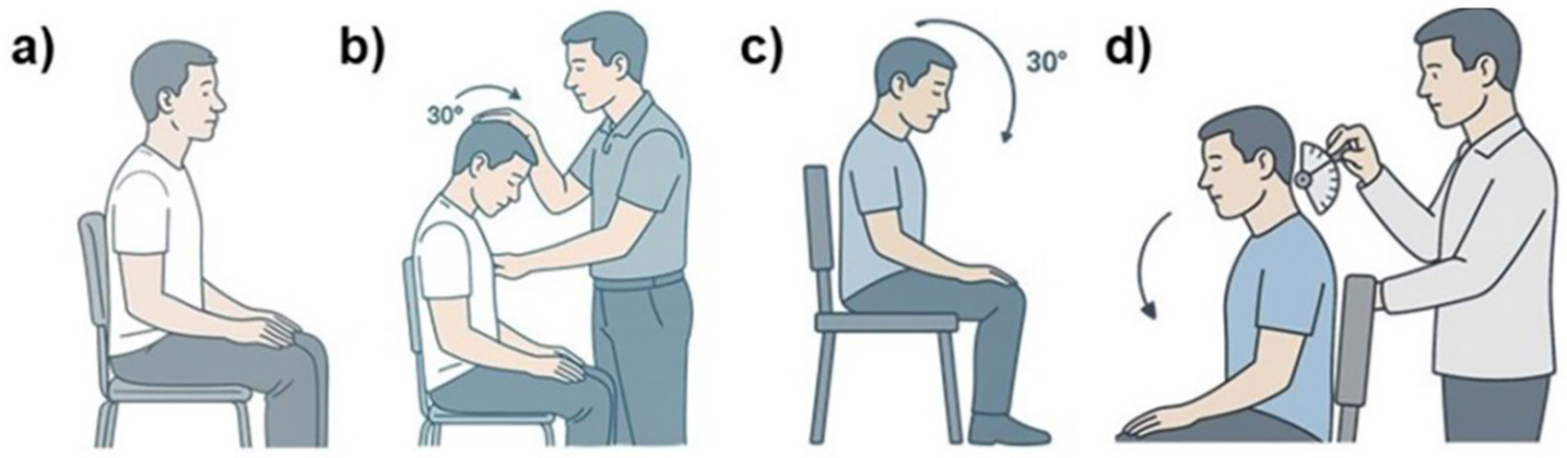
Head repositioning accuracy (HRA) assessment (cervicocephalic relocation test) in the sagittal plane. **(a)** Person sits upright, **(b)** examiner flexes person’s neck 30 degrees forward, **(c)** person sits with neck maintained at 30-degree flexion, and **(d)** examiner uses reflex hammer on person’s neck while flexed.

Following this passive learning phase, participants were asked to actively reproduce the learned movement, still with eyes closed, attempting to accurately reach the same 30° flexion position. Once the participant signaled that the target position had been reached, the evaluator measured the angular difference between the target and the reproduced position using a universal goniometer, recording the absolute angular error in degrees (See Figure 1).

Cervical motor control was assessed by a single trained evaluator who followed a standardized testing protocol for all participants. For each movement direction (flexion, extension, left rotation, and right rotation), participants performed one active repositioning attempt following the passive learning phase, and the absolute angular error was recorded in degrees. Short rest periods were allowed between movements to minimize muscular fatigue. Although intra- and inter-rater reliability were not formally assessed in the present study, the Head Repositioning Accuracy-to-Target test has demonstrated acceptable reliability and validity in previous research (Michiels et al., 2013).

### 2.5 Statistical analysis

Statistical analysis was performed using IBM SPSS Statistics software (version 28). A statistical significance level of *p* < 0.05 was established for all tests.

#### 2.5.1 Preliminary and descriptive analysis

The distribution of quantitative variables was explored using Kolmogorov- Smirnov normality tests. Since several of the variables did not follow normal distribution, we chose to use non-parametric tests for inferential analyses.

Descriptive measures (means, standard deviations, frequencies and percentages) were calculated to characterize the sample and to describe the levels of anxiety, pain and cervical motor control. Comparisons between groups according to the degree of anxiety (mild, moderate, severe) were also performed using descriptive analyses.

#### 2.5.2 Relationships between variables

We used Spearman’s correlation coefficient to evaluate the relationship between quantitative variables, i.e., total score on the Hamilton Anxiety Scale, angular error in cervical motor control and NRS pain scales, and Pearson’s chi-square test to establish possible associations between qualitative variables, such as sex, vertigo, headache, cervical pain or temporomandibular dysfunction and degree of anxiety.

#### 2.5.3 Multivariate analysis

To explore factors associated with anxiety, two multivariate analysis approaches were applied:

##### 2.5.3.1 Multiple linear regression

A multiple linear regression model was constructed to identify potential quantitative associations with anxiety levels, using the total score on the Hamilton Anxiety Scale (HAM-A) as the dependent variable. The independent variables included angular errors in cervical movements—specifically in flexion, extension, and right and left rotation—as well as clinical variables such as vertigo, pain, and temporomandibular dysfunction, and sociodemographic factors including age, sex, weight, and height. The analysis was conducted using the forced-entry method (enter), which allows for the simultaneous inclusion of all selected predictors. Prior to interpreting the results, the assumptions of normality, homoscedasticity, linearity, and independence of residuals were verified. Additionally, the absence of multicollinearity was examined through the variance inflation factor (VIF). The overall fit of the model was evaluated using the coefficient of determination (R^2^), and the statistical significance of the model was assessed via the F test from analysis of variance (ANOVA).

##### 2.5.3.2 Binary logistic regression

To identify associations with clinically significant anxiety, the HAM-A variable was recoded into a dichotomous outcome, where a score of 0 indicated mild or normal anxiety (HAM-A ≤ 17), and a score of 1 represented moderate to severe anxiety (HAM-A ≥ 18). A binary logistic regression model was then applied, using the same set of predictors as in the linear regression model. The overall quality of the model fit was evaluated using the chi-square likelihood ratio statistic and Nagelkerke’s pseudo-R^2^. For each predictor, odds ratios (Ors) were calculated along with their corresponding 95% confidence intervals. In addition, the model’s sensitivity, specificity, and overall classification accuracy were reported. Finally, multicollinearity and goodness of fit were assessed, and when applicable, the Hosmer-Lemeshow test was used to evaluate model calibration.

Both regression approaches were conducted with an exploratory aim, to examine associations between anxiety symptoms and cervical sensorimotor variables, rather than to develop predictive or diagnostic models.

Residual diagnostics (standardized residual distribution, skewness, and kurtosis) were evaluated only for the linear regression model, as normality and homoscedasticity assumptions apply to linear models but are not required for logistic regression.

#### 2.5.4 Covariates

Several covariates were collected for descriptive purposes and to control for potential confounding factors in multivariate statistical analyses. These included age, sex, height, and weight.

Physical activity level was also registered using a self-administered digital questionnaire, where participants reported the number of days per week and duration of any physical activity. Additionally, self-reported clinical variables were collected, including the presence of dizziness, headaches, cervical pain, and limitations in cervical movement. For both headaches and cervical pain, information was gathered with regards to the type and intensity of pain. For the latter we employed a visual analog scale (VAS) developed by Gallagher et al. (2002). In this study, we applied a simplified numerical version of the VAS, where participants were asked to rate their pain on a scale from 0 to 10, where 0 represents “no pain” and 10 indicates “the worst pain imaginable.” This version, also referred to as the Numeric Rating Scale (NRS), still shows strong psychometric properties, with high validity and reliability, and is particularly useful in busy clinical and non-clinical settings as it facilitates administration (Safikhani et al., 2018).

## 3 Results

### 3.1 Sociodemographic and clinical characteristics

The sample included 101 participants, with a mean age of 26.4 years. Female sex was more prevalent (64.4%). Regarding clinical variables, the prevalence of cervical pain (45.5%), temporomandibular dysfunction (43.0%), vertigo (35.6%), and headache (37.6%) was recorded. In the cervical motor control tests, mean angular error values ranged above 4° across all evaluated movements. The mean score on the Hamilton Anxiety Rating Scale was 8.95 points, with higher values observed in the psychological symptom dimension (see Table 1).

**TABLE 1:** Characteristics of the study participants.

Variable	Mean (standard deviation)/count (percentage)
Age (years)	26.4 (9.6)
Sex (% women)	65 (64.4%)
Weight (kg)	67.5 (11.3)
Height (cm)	168.6 (7.9)
Presence of vertigo (%)	36 (35.6%)
Headache (%)	38 (37.6%)
Cervical pain (%)	46 (45.5%)
Temporomandibular disorders (%)	43 (43.0%)
Angular error in flexion (°)	4.73 (2.9)
Angular error in extension (°)	4.5 (2.1)
Angular error in left rotation (°)	4.98 (2.6)
Angular error in right rotation (°)	4.84 (2.6)
NRS headache (points)	2.04 (2.7)
NRS cervical pain (points)	1.74 (2.2)
Total HAM-A (points)	8.95 (7.1)
Psychic HAM-A (points)	5.5 (4.6)
Somatic HAM-A (points)	3.46 (3.2)

HAM, Hamilton Anxiety Rating Scale; NRS, Numeric Rating Scale.

### 3.2 Association of cervical motor control and anxiety symptoms

The final sample was classified into three groups according to anxiety severity based on the HAM-A score: mild (*n* = 86), moderate (*n* = 11), and severe anxiety (*n* = 4). Table 2 presents the descriptive characteristics of sociodemographic, clinical, and functional variables for each group.

**TABLE 2:** Characteristics of the study participants according to their anxiety level.

Variable	Mild anxiety (*n* = 86)	Moderate anxiety (*n* = 11)	Severe anxiety (*n* = 4)
Age (years)	25.42 ± 8.83	32.36 ± 12.09	31.00 ± 13.98
Flexion (°)	3.94 ± 2.21	8.91 ± 1.87	10.25 ± 1.50
Extension (°)	4.02 ± 1.86	7.09 ± 1.38	7.50 ± 0.58
Left rotation (°)	4.53 ± 2.41	7.18 ± 1.40	8.50 ± 1.73
Right rotation (°)	4.31 ± 2.36	7.82 ± 1.25	8.00 ± 2.16
NRS head pain (points)	1.81 ± 2.54	2.36 ± 2.87	6.00 ± 2.16
NRS cervical pain (points)	1.34 ± 1.93	3.36 ± 2.11	6.00 ± 2.31
Psychic HAM (points)	4.12 ± 3.14	12.18 ± 2.14	16.75 ± 2.63
Somatic HAM (points)	2.51 ± 2.23	8.55 ± 2.42	9.75 ± 2.22
Total HAM (points)	6.63 ± 4.55	20.73 ± 2.28	26.50 ± 1.29
Sex (female)	54 (62.8%)	7 (63.6%)	4 (100%)
Vertigo (yes)	25 (29.1%)	7 (63.6%)	4 (100%)
Headache (yes)	29 (33.7%)	5 (45.5%)	4 (100%)
Cervical pain (yes)	33 (38.4%)	9 (81.8%)	4 (100%)
Mandibular alterations (yes)	34 (40.0%)	7 (63.6%)	2 (50%)

HAM, Hamilton Anxiety Rating Scale; NRS, Numeric Rating Scale. Subgroup comparisons involving moderate and severe anxiety levels should be interpreted descriptively due to small sample sizes.

Regarding sociodemographic variables, participants in the moderate and severe anxiety groups showed higher mean age values (32.4 and 31.0 years, respectively) compared with the mild anxiety group (25.4 years). The proportion of females was high across all groups, reaching 100% in the severe anxiety group.

For cervical motor control, higher mean angular error values were observed in the moderate and severe anxiety groups compared with the mild group. In the mild anxiety group, angular error values ranged between 3.9 and 4.5°, whereas in the moderate and severe groups, values ranged between 7 and 10° across the different movements evaluated (flexion, extension, left rotation, and right rotation).

Pain perception, assessed using the NRS scale, showed higher mean values in the moderate and severe anxiety groups compared with the mild group. Participants with mild anxiety reported mean headache and neck pain scores of 1.81 and 1.34, respectively, whereas participants with severe anxiety showed mean values of six points for both locations.

Regarding anxiety dimensions, mean scores for the psychological and somatic subscales of the HAM-A were higher in the moderate and severe anxiety groups than in the mild anxiety group. The psychological subscale ranged from from a mean of 4.12 in the mild group to 16.75 in the severe group, while the somatic subscale ranged from 2.51 to 9.75.

Descriptive values for cervical motor control, pain perception, and anxiety dimensions across anxiety groups are presented in Table 2.

### 3.3 Relationships between anxiety, pain and cervical motor control

Correlational analyses identified statistically significant associations between anxiety severity and several clinical and functional variables. Higher HAM-A scores were associated with higher angular error values across all cervical movements evaluated. The largest correlations were observed for cervical flexion (ρ = 0.80; *p* < 0.001) and extension (ρ = 0.73; *p* < 0.001).

Anxiety severity was also associated with pain perception. HAM-A total scores showed a moderate correlation with cervical pain intensity (ρ = 0.47; *p* < 0.001) and a weaker correlation with headache intensity (ρ = 0.23; *p* = 0.021).

When anxiety dimensions were analyzed separately, the psychological subscale of the HAM-A showed higher correlation coefficients with cervical motor control variables than the somatic subscale. For cervical flexion, correlation coefficients were ρ = 0.76 for the psychological dimension and ρ = 0.68 for the somatic dimension (both *p* < 0.001). Similar patterns were observed across the remaining cervical movements, including extension and right rotation.

Cervical pain intensity showed comparable correlations with the psychological (ρ = 0.47) and somatic (ρ = 0.46) dimensions of anxiety (both *p* < 0.001). In contrast, headache intensity was significantly correlated with the somatic dimension (ρ = 0.25; *p* = 0.010), whereas no statistically significant correlation was observed with the psychological dimension.

Additionally, angular error values in cervical motor control were associated with cervical pain intensity, particularly in flexion (ρ = 0.46; *p* < 0.001) and extension (ρ = 0.36; *p* < 0.001). Headache intensity showed a statistically significant association only with angular error in cervical flexion (ρ = 0.21; *p* = 0.043).

Correlation coefficients for anxiety severity, cervical motor control variables, and pain perception are presented in Supplementary Table 1.

### 3.4 Exploratory association models

#### 3.4.1 Multiple linear regression: factors associated with anxiety severity

A multiple linear regression analysis was conducted to explore the association between clinical and sensorimotor variables and the total score on the HAM-A. The model included all variables assessed in the study.

The model was statistically significant [F(14, 85) = 29.03; *p* < 0.001] and accounted for a relatively large proportion of variance within this sample in anxiety scores (R^2^ = 0.827; adjusted R^2^ = 0.799), within an exploratory analytical framework. Among the variables included, errors in cervical flexion, extension, and right rotation, as well as the presence of vertigo, showed statistically significant associations with the HAM-A total score (see Supplementary Table 2).

Other variables, including age, sex, cervical pain, and temporomandibular dysfunction, did not reach statistical significance in the model. No evidence of problematic multicollinearity was observed (all VIF < 3.3), and inspection of residuals indicated an approximately normal and homoscedastic distribution (see Supplementary material).

#### 3.4.2 Binary logistic regression: exploratory association with anxiety severity

A binary logistic regression analysis was conducted to explore the association between clinical and sensorimotor variables and anxiety severity categorized according to the HAM-A score (0 = mild or absent anxiety; 1 = moderate or severe anxiety, HAM-A ≥ 18). All study variables were included in the model.

The model was statistically significant [χ^2^(4) = 76.65; *p* < 0.001] and accounted for a relatively large proportion of variance within this sample (Nagelkerke R^2^ = 0.753), within an exploratory analytical framework. Errors in cervical flexion, extension, and rotation, as well as the presence of vertigo, showed statistically significant associations with the dichotomized anxiety variable (see Supplementary Table 3).

Other variables included in the model did not reach statistical significance. Results from the logistic regression are presented descriptively and should be interpreted as exploratory associations rather than as indicators of diagnostic or predictive performance.

## 4 Discussion

The present study explored the association between anxiety symptoms and cervical motor control in individuals without diagnosed psychiatric or neurological disorders. The findings suggest that higher levels of anxiety are associated with poorer cervical motor control performance, particularly in flexion, extension, and right rotation movements, as well as with increased pain perception and the presence of vertigo. Importantly, these associations were observed in a non-clinical sample and should be interpreted within the context of the study’s cross-sectional and exploratory design.

The present findings indicate an association between higher levels of anxiety symptoms and poorer cervical motor control, as well as increased pain perception in the cervical region. These results are consistent with previous research reporting an overlap between anxiety, musculoskeletal symptoms, and altered motor control (Blozik et al., 2009; Kazeminasab et al., 2022). Although the underlying mechanisms cannot be determined from the present cross-sectional design, one possible interpretation is that anxiety-related changes in central nervous system processing may influence sensorimotor integration and motor coordination. Previous studies have suggested that emotional states can modulate posture and motor control through cortical and subcortical mechanisms affecting proprioception and muscle tone (D’Attilio et al., 2013; Hall et al., 2023).

Likewise, an association was observed between the presence of vertigo and higher anxiety severity. This is consistent with previous studies that pointed out high comorbidity between vestibular symptoms and affective disorders (Chen et al., 2024). Although the mechanisms underlying this association cannot be established in the present study, previous research has suggested that shared neural circuits between the vestibular system and brain regions involved in emotional regulation, such as the insula, amygdala, and hippocampus, may contribute to this relationship (Neumann et al., 2023).

In addition, differential associations were observed between specific dimensions of anxiety and related clinical symptoms. Participants reporting vertigo exhibited higher levels of psychological anxiety, characterized by symptoms such as intrusive thoughts, excessive worry, and insomnia (Eckhardt et al., 1996). This may suggest that cognitive-emotional processes are involved in certain vestibular complaints, potentially through mechanisms related to autonomic and/or subcortical dysfunction. Conversely, the intensity of headache was strongly correlated with somatic symptoms of anxiety, such as tremors, sweating, or chest tightness (Cengiz et al., 2019).

### 4.1 Clinical and scientific implications

From a research perspective, the present findings should be considered hypothesis-generating rather than directly applicable. The observed associations between anxiety symptoms and cervical motor control in a non-clinical sample suggest that emotional factors may be related to subtle sensorimotor alterations beyond pain-related mechanisms. These results contribute to a growing body of literature highlighting the close interaction between emotional states and motor control processes and may inform future longitudinal or interventional studies aimed at clarifying the temporal dynamics and clinical relevance of these associations.

More broadly, the involvement of motor control and somatic perception is consistent with contemporary models that conceptualize anxiety as a multisystemic phenomenon involving dynamic interactions between central and peripheral processes, such as those described within interoceptive framework (Craig, 2002). Further research is needed to determine whether these sensorimotor alterations precede, follow, or interact bidirectionally with anxiety symptoms across different populations.

### 4.2 Limitations

This study has several limitations that should be considered when interpreting the results. First, the cross-sectional design precludes causal inference regarding the direction of the observed associations between anxiety symptoms and cervical motor control. Therefore, it cannot be determined whether anxiety contributes to sensorimotor alterations, whether motor dysfunction influences anxiety symptoms, or whether both are influenced by other unmeasured factors.

Second, the study was conducted in a non-clinical sample without diagnosed psychiatric or neurological disorders, composed predominantly of young adults, which may limit the generalizability of the findings to other populations. In addition, the sample size, particularly within the moderate and severe anxiety subgroups, was relatively small, which may affect the stability of the multivariate models.

The inclusion of correlated cervical motor control variables within the same regression models may have contributed to an overestimation of the explained variance. Similarly, the dichotomization of anxiety severity, together with the small size of the moderate/severe anxiety subgroup, may have inflated classification metrics and affected model stability, particularly in the logistic regression analyses. Consequently, these regression models should be interpreted within an exploratory and hypothesis-generating framework rather than as predictive or screening tools. The high proportion of explained variance observed should therefore be interpreted cautiously, as it may partly reflect model complexity, correlated predictors, and the exploratory nature of the analyses. Moreover, no internal validation procedures (e.g., bootstrapping or cross-validation) were performed, and future studies with larger samples are needed to assess the robustness and generalizability of these associations.

Furthermore, we did not control external factors that could have had an influence on the results, such as sleep quality, academic stress, or level of physical activity. Finally, although the measures we used to evaluate cervical motor control were objective, other techniques such as surface electromyography or functional neuroimaging could have allowed us to explore the underlying neurophysiological mechanisms in greater depth and should be considered in future research.

### 4.3 Futures lines of research

Longitudinal and prospective studies are essential to determine whether alterations in cervical motor control are a cause, consequence, or maintaining factor of anxiety, with prospective designs and mediation approaches helping to clarify the directionality of these associations. In addition, experimental interventions, including controlled interventional studies, could help explore whether improvements in cervical motor control are associated with changes in anxiety symptoms. Validation of these findings in clinical populations, such as individuals with anxiety disorders, would be important to examine their relevance beyond subclinical contexts. Finally, studies using neuroimaging or functional connectivity techniques can help elucidate how somatosensory, vestibular, and limbic networks interact with emotional processing, providing neurobiological support for the functional observations reported.

## 5 Conclusion

This study found an association between anxiety symptoms and cervical motor control alterations in individuals without diagnosed psychiatric or neurological disorders. Specifically, greater sensorimotor impairments—particularly in flexion, extension, and right rotation—were associated with higher levels of anxiety. These findings, derived from a cross-sectional and exploratory design, highlight the need for further longitudinal research to clarify the direction and significance of this relationship.

## Data Availability

The original contributions presented in this study are included in this article/Supplementary material, further inquiries can be directed to the corresponding author.
