# Supplementary material for: Relationship between anxiety symptoms and cervical motor control in individuals without diagnosed psychiatric or neurological disorders
Source: Front Psychol. 2026 Feb 25;17:1743293. doi: 10.3389/fpsyg.2026.1743293 (PMC12975477; doi:10.3389/fpsyg.2026.1743293)

**Supplementary Figure S2: Normal Q–Q plot of standardized residuals from the multiple linear regression model.** Visual assessment of the normality assumption of model residuals.
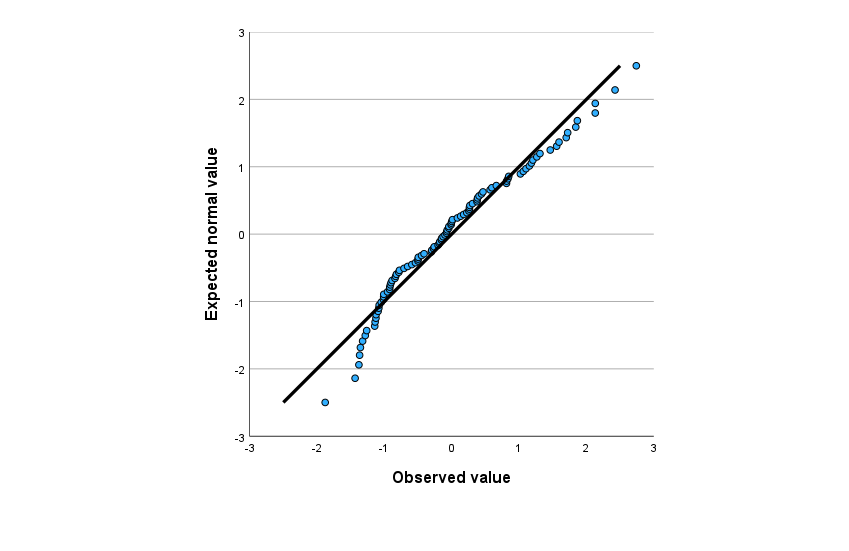

Supplement: Supplementary file 1 [file Data_Sheet_1.zip › 1743293_Data_Sheet_1/Figure 2.docx]
