# Supplementary material for: Relationship between anxiety symptoms and cervical motor control in individuals without diagnosed psychiatric or neurological disorders
Source: Front Psychol. 2026 Feb 25;17:1743293. doi: 10.3389/fpsyg.2026.1743293 (PMC12975477; doi:10.3389/fpsyg.2026.1743293)

**Supplementary Figure S6: Scatterplot of left cervical rotation angular error versus total HAM-A score** All individual observations are displayed. The solid line represents the fitted linear regression model, and the two additional lines indicate the upper and lower limits of the 95% confidence interval.


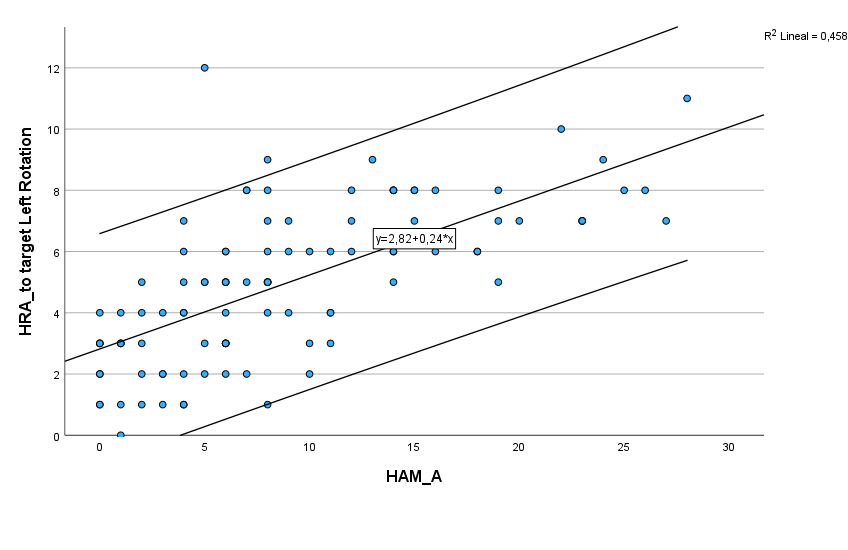

Supplement: Supplementary file 1 [file Data_Sheet_1.zip › 1743293_Data_Sheet_1/Figure 6.docx]
