# Supplementary material for: Relationship between anxiety symptoms and cervical motor control in individuals without diagnosed psychiatric or neurological disorders
Source: Front Psychol. 2026 Feb 25;17:1743293. doi: 10.3389/fpsyg.2026.1743293 (PMC12975477; doi:10.3389/fpsyg.2026.1743293)
Supplement: Supplementary file 1 [file Data_Sheet_1.zip › 1743293_Data_Sheet_1/Table 1.DOCX]

**Supplementary Table 1.** Spearman correlation coefficients (ρ) with 95% confidence intervals (CI) between anxiety, pain, and cervical motor control.

| **Variable** | **Total**  **HAM ρ (95% CI)** | **Psychic HAM ρ (95% CI)** | **Somatic HAM ρ (95% CI)** | **Cervical pain ρ (95% CI)** | **Headache ρ (95% CI)** |
| --- | --- | --- | --- | --- | --- |
| Flexion (°) | 0.797 (0.710–0.860)** | 0.756 (0.654–0.831)** | 0.678 (0.552–0.773)** | 0.459 (0.284–0.604)** | 0.201 (0.000–0.387)* |
| Extension (°) | 0.731 (0.622–0.813)** | 0.723 (0.610–0.806)** | 0.574 (0.422–0.695)** | 0.356 (0.167–0.520)** | −0.004 (−0.205–0.197) |
| Left rotation (°) | 0.718 (0.604–0.803)** | 0.648 (0.514–0.751)** | 0.658 (0.527–0.759)** | 0.278 (0.081–0.453)** | 0.075 (−0.128–0.272) |
| Right rotation (°) | 0.776 (0.681–0.845)** | 0.711 (0.595–0.798)** | 0.681 (0.556–0.776)** | 0.276 (0.080–0.452)** | 0.085 (−0.118–0.281) |
| NRS Headache (points) | 0.229 (0.029–0.411)* | 0.189 (−0.012–0.376) | 0.254 (0.056–0.433)* | 0.371 (0.183–0.532)** | — |
| NRS Cervical pain (points) | 0.466 (0.292–0.610)** | 0.455 (0.280–0.601)** | 0.365 (0.176–0.527)** | — | — |

* p < 0.05. ** p <0.01. p=Spearman’s coefficients. HAM: Hamilton Anxiety Rating Scale. NRS: Numeric Rating Scale. 95% confidence intervals were estimated using Fisher’s r-to-z transformation. Standard error was calculated according to the method proposed by Fieller, Hartley and Pearson.
