# Supplementary material for: Relationship between anxiety symptoms and cervical motor control in individuals without diagnosed psychiatric or neurological disorders
Source: Front Psychol. 2026 Feb 25;17:1743293. doi: 10.3389/fpsyg.2026.1743293 (PMC12975477; doi:10.3389/fpsyg.2026.1743293)
Supplement: Supplementary file 1 [file Data_Sheet_1.zip › 1743293_Data_Sheet_1/Table 2.DOCX]

**Supplementary Table 2.** Multivariable linear regression model predicting total anxiety score (HAM-A) using the enter method.

| **Predictor variable** | **Standardized coefficient (β)** | **95% confidence interval** | **t-value** | **p-value** |
| --- | --- | --- | --- | --- |
| Error in cervical flexion | 0.332 | (0.43-1.20) | 4.227 | < 0.001 |
| Error in cervical extension | 0.234 | (0.34-1.24) | 3.501 | < 0.001 |
| Error in left rotation | 0.073 | (-0.19-0.60) | 1.031 | 0.305 |
| Error in right rotation | 0.255 | (0.31-1.11) | 3.493 | < 0.001 |
| Presence of vertigo | 0.110 | (0.09-3.16) | 2.100 | 0.039 |
| Cervical pain | 0.112 | (-0.21-0.92) | 1.251 | 0.214 |
| Headache | 0.171 | (-0.09-1.00) | 1.676 | 0.097 |
