# Supplementary material for: Relationship between anxiety symptoms and cervical motor control in individuals without diagnosed psychiatric or neurological disorders
Source: Front Psychol. 2026 Feb 25;17:1743293. doi: 10.3389/fpsyg.2026.1743293 (PMC12975477; doi:10.3389/fpsyg.2026.1743293)
Supplement: Supplementary file 1 [file Data_Sheet_1.zip › 1743293_Data_Sheet_1/Table 3.DOCX]

**Supplementary table 3.** Comparative summary of models.

|  | Linear model fit | | | Logistic model fit | | | | |
| --- | --- | --- | --- | --- | --- | --- | --- | --- |
| **Predictor** | **B** | **SE** | **p** | **B** | **SE** | **OR** | **95% CI OR** | **p** |
| **Constant** | 0.088 | 9.611 | 0.993 | -16.266 | 13.906 | — | — | 0.242 |
| **Cervical flexion** | 0.848 | 0.201 | <0.001 | 0.438 | 0.245 | 1.550 | 1.183 – 1.845 | 0.073 |
| **Cervical extension** | 0.786 | 0.233 | 0.001 | 0.219 | 0.375 | 1.245 | 1.064 – 1.748 | 0.559 |
| **Cervical right rotation** | 0.704 | 0.208 | 0.001 | 0.032 | 0.332 | 1.032 | 1.135 – 1.749 | 0.924 |
| **Vertigo (yes)** | 2.123 | 0.906 | 0.021 | 2.024 | 1.163 | 7.570 | 1.451 – 8.859 | 0.082 |

*Linear model fit*: R² = 0.827; Adjusted R² = 0.799; F (14,85) = 29.034; p < 0.001. N = 100. *Logistic model fit*: −2 Log Likelihood = 41.159; Cox & Snell R² = 0.329; Nagelkerke R² = 0.592; Omnibus χ²(13) = 39.834, p < 0.001. Events = 14/100; Events-per-variable ratio (EPV) = 1.07.
