# Supplementary material for: Relationship between anxiety symptoms and cervical motor control in individuals without diagnosed psychiatric or neurological disorders
Source: Front Psychol. 2026 Feb 25;17:1743293. doi: 10.3389/fpsyg.2026.1743293 (PMC12975477; doi:10.3389/fpsyg.2026.1743293)
Supplement: Supplementary file 1 [file Data_Sheet_1.zip › 1743293_Data_Sheet_1/Table 4.docx]

**Supplementary Table S4: Diagnostic assessment of standardized residuals from the multivariable linear regression model.** Min = minimum; Max = maximum; Mean = arithmetic mean; SD = standard deviation. Skewness and kurtosis values are reported together with their standard errors (SE).

|  | Min | Max | Mean | SD | Swekness | | Kurtosis | |
| --- | --- | --- | --- | --- | --- | --- | --- | --- |
|  |  |  |  |  | *Value* | *SE* | *Value* | *SE* |
| Standarized residual | -1.83 | 2.67 | 0.00 | 0.93 | 0.42 | 0.24 | 0.37 | 0.48 |
